# Glucocorticoid mediates prenatal caffeine exposure-induced endochondral ossification retardation and its molecular mechanism in female fetal rats

**DOI:** 10.1038/cddis.2017.546

**Published:** 2017-10-26

**Authors:** Yangfan Shangguan, Hongqiang Jiang, Zhengqi Pan, Hao Xiao, Yang Tan, Kai Tie, Jun Qin, Yu Deng, Liaobin Chen, Hui Wang

**Affiliations:** 1 Department of Orthopedic Surgery, Zhongnan Hospital of Wuhan University, Wuhan 430071, China; 2 Hubei Provincial Key Laboratory of Developmentally Originated Disease, Wuhan 430071, China; 3 Department of Pharmacology, Basic Medical School of Wuhan University, Wuhan 430071, China

## Abstract

Our previous studies discovered that prenatal caffeine exposure (PCE) could induce intrauterine growth retardation (IUGR) and long-bone dysplasia in offspring rats, accompanied by maternal glucocorticoid over-exposure. This study is to explore whether intrauterine high glucocorticoid level can cause endochondral ossification retardation and clarify its molecular mechanism in PCE fetal rats. Pregnant Wistar rats were intragastrically administered 30 and 120 mg/kg day of caffeine during gestational days (GDs) 9–20, then collected fetal serum and femurs at GD20. *In vitro*, primary chondrocytes were treated with corticosterone (0–1250 nM), caffeine (0–100 *μ*M), mitogen-inducible gene 6 (Mig-6) siRNA and epidermal growth factor receptor (EGFR) siRNA, respectively, or together. Results showed that the hypertrophic chondrocytes zone (HZ) of PCE fetal femur was widened. Meanwhile, the expression levels of chondrocytes terminal differentiation genes in the HZ were decreased, and the chondrocytes apoptosis rate in the HZ was decreased too. Furthermore, PCE upregulated Mig-6 and suppressed EGFR expression in the HZ. *In vitro*, a high-concentration corticosterone (1250 nM) upregulated Mig-6 expression, inhibit EGFR/c-Jun N-terminal kinase (JNK) signaling pathway and terminal differentiation genes expression in chondrocytes and reduced cell apoptosis, and these above alterations could be partly reversed step-by-step after Mig-6 and EGFR knockdown. However, caffeine concentration dependently increased chondrocyte apoptosis without significant changes in the expression of terminal differentiation genes. Collectively, PCE caused endochondral ossification retardation in the female fetal rats, and its main mechanism was associated with glucocorticoid (rather than caffeine)-mediated chondrocyte terminal differentiation suppression by the upregulation of Mig-6 and then inhibition of EGFR/JNK pathway-mediated chondrocyte apoptosis.

Recent years, increasing research has focused on the effects of the intrauterine growth environment on adult heath and disease. Barker’s theory proposed that infants with low birthweights are more vulnerable to metabolic syndrome (MS) in adult life, hypothesizing the fetal origin of adult diseases.^1^ In the last decade, the developmental origin of health and disease (DOHaD), widely known as early-life development, has been found to have important effects on later life. Caffeine (1,3,7-trimethylxanthine), a plant alkaloid found in coffee, tea, cocoa and cola soft drinks, is one of the most frequently used substances with pharmacological properties in the world.^2^ However, caffeine’s safety problems have always been the emphasis of people’s concern. Epidemiological and animal experiments indicate that caffeine can cause reproductive and developmental toxicities, including intrauterine growth retardation (IUGR).^3, 4^ IUGR, mainly presented as a low birthweight, is associated with increased susceptibility to adult osteoporosis.^5^ With postmenopausal osteoporosis occurring, women are more likely to suffer from osteoporosis than men.^6^ Epidemiological studies found that the morbidity of osteoporosis in women was higher than in men, and older women have a high probability of osteoporotic hip fracture,^7^ which suggested that osteoporosis is more worthy of attention in women. Thus, the influence of prenatal caffeine ingestion on female fetal bone development was of interest. Our previous studies demonstrated that prenatal caffeine exposure (PCE) can induce IUGR and growth plate retardation in female fetal rats,^8^ and we proposed that the underlying mechanism probably related with over-exposure to a high level of maternal glucocorticoid.^9^

Basic glucocorticoids (humans as cortisol, rodents as corticosterone) are crucial to controlling fetal bone histomorphology and functional maturity.^10^ Nevertheless, maternal long-term use of dexamethasone in pregnancy and excessive endogenous glucocorticoid exposure can bring about abnormal fetal bone development.^11, 12^ The main developmental form of long-bone formation during the embryotic period is endochondral ossification. Mesenchymal cells assemble and form the cartilage prototype, followed by chondrocyte proliferative, differentiation and apoptosis. Terminally differentiated chondrocytes secrete multiple growth factors to promote ossification center formation, also provide space for forming an ossification center.^13^ The epidermal growth factor receptor (EGFR) signal participates in survival, adhesion, proliferative, migration and differentiation of various bone cells.^14^ Previous studies have reported that EGFR deficient mice have delayed primary endochondral ossification accompanied by broadening of the cartilage growth plate of the hypertrophy zone (HZ).^15^ The EGFR signal may take part in regulating terminal differentiation of the hypertrophy of chondrocytes in the endochondral ossification process. Mitogen-inducible gene 6 (Mig-6) is an early immediate gene, and it has important roles in regulating the reaction to stress.^16^ Researchers found that Mig-6 can be recruited and bind to the EGFR kinase domains, thus inhibiting its phosphorylation and downstream signal activity.^17^ Meanwhile, the researchers also found that a high concentration of glucocorticoid suppress the EGFR signal, which is closely relevant to Mig-6 upregulation in various cells and tissues.^18^ In summary, we supposed that PCE-induced fetal over-exposure to maternal glucocorticoid probably upregulate Mig-6 and then suppress the EGFR signal, thus affecting the terminal differentiation of chondrocytes in the endochondral ossification process during embryonic development.

On the basis of our established rat IUGR model by PCE,^19^ the aim of this study is to explore whether a high glucocorticoid level can cause endochondral ossification retardation, and to clarify its molecular mechanism in the PCE female fetal rats. This study will help to explain pregnant adverse environments-induced long-bone developmental toxicity and the intrauterine origin of adult osteoporosis.

## Results

### Effects of PCE on the femur length and histology of fetal rats

**Figure 1:**
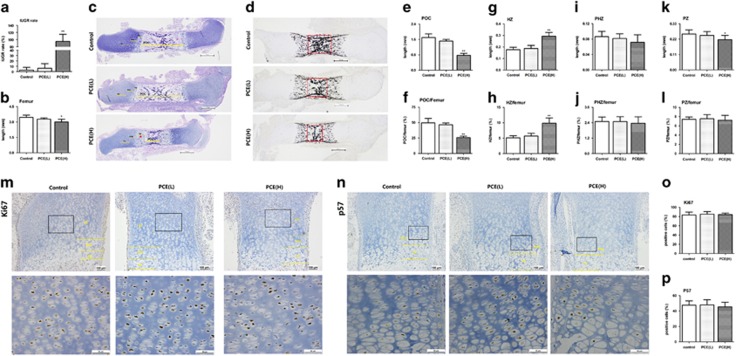
Effects of PCE on fetal femurs histology, variations of proliferation and differentiation. (**a**) IUGR rate. (**b**) Length of femur (mm). (**c**) H&E staining of fetal femur. (**d**) Von Kossa staining of fetal femur. (**e**) Length of primary ossification center (POC) (mm). (**f**) POC/femur (%). (**g**) Length of hypertrophic chondrocytes zones (HZ) (mm). (**h**) HZ/femur (%). (**i**) Length of pre-hypertrophic chondrocytes zones (PHZ) (mm). (**j**) PH/femur (%). (**k**) Length of proliferative chondrocytes zones (PZ) (mm). (**l**) PZ/femur (%). (**m**) Immunostaining of Ki67. (**n**) Immunostaining of p57. (**o**) Quantification of Ki67 (positive rate). (**p**) Quantification of P57 (positive rate). *n*=5 per group obtained from different litters. Three random fields/section for quantitative. Data are shown as the mean±S.D. *P<0.05, **P<0.01 *versus* control (ANOVA)

To confirm the effect of PCE on long-bone development of female fetal rats, caffeine at 30 and 120 mg/kg day was intragastrically administered from GD9 to GD20. The fetal IUGR rate was calculated and the femurs were harvest for histological analysis at GD20. We found that PCE(H) group has a significantly increased IUGR rate and a shorter femur length compared with control group (*P*<0.01, Figures 1a–c). Further analysis showed that, the absolute and relative lengths of POC in the PCE(H) group were shorter than those of the control (*P*<0.01, Figures 1c, e and f), and the number of mineralized nodules in the POC of PCE(H) fetal rats was sparser than that of the control (Figure 1d). Meanwhile, the absolute and relative lengths of the HZ in the PCE(H) group were longer than those of the control (*P*<0.01, Figures 1g and h), but the lengths of pre-hypertrophic zone (PHZ) and proliferation zone (PZ) had no statistical difference between control group and PCE groups (Figures 1i–l). Immunohistochemistry (IHC) analyse for Ki67 in PZ and P57 expression in the PHZ also showed no statistical difference (Figures 1m–p). Therefore, these results suggest that the PCE delays long-bone development in the female fetal rats, which might be associated with the broadening of the HZ.

### Effects of PCE on terminal differentiation and apoptosis in the HZ of fetal rats

**Figure 2:**
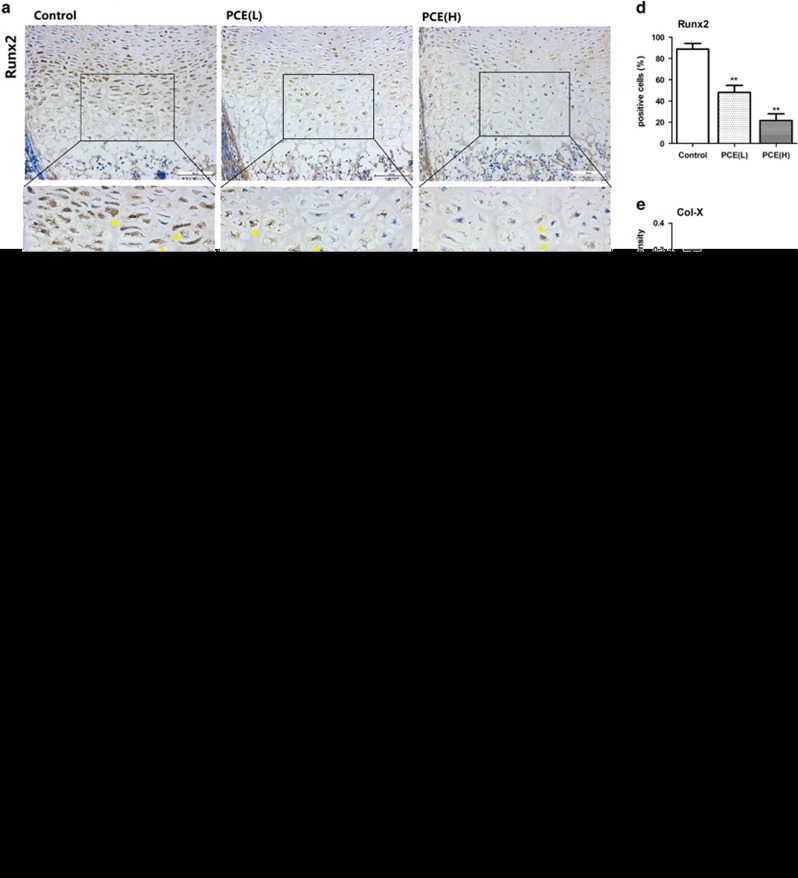
Effects of prenatal caffeine expose (PCE) on terminal differentiation of hypertrophic chondrocytes in fetal long bone. (**a**) Immunostaining of Runt-related transcription factor 2 (Runx2) in hypertrophic chondrocytes. (**b**) *In situ* hybridization (ISH) of collagen type X (Col-X) in hypertrophic chondrocytes. (**c**) Fluorescent TdT-mediated dUTP nick-end labeling (TUNEL) staining in hypertrophic chondrocytes. (**d**) Quantification of Runx2 (positive rate). (**e**) Quantification of Col-X ISH (fluorescence intensity). (**f**) Quantification of fluorescent TUNEL (positive rate). *n*=5 per group obtained from different litters. Three random fields/section for quantitative. Data are shown as the mean±S.D. *P<0.05, **P<0.01 *versus* control (ANOVA)

Furthermore, our study focused on the terminal differentiation and apoptosis in the HZ of the fetal femur as it had significant changes in the PCE groups. The IHC analyses showed that the expression of Runx2 in the HZ of PCE(L) and PCE(H) fetal femur were decreased in the dose-dependent manners (*P*<0.01, Figures 2a and d). *In situ* hybridization (ISH) results showed that the expression of Col-X between control and PCE(H) groups has statistical difference (*P*<0.05, Figures 2b and e). Fluorescent TUNEL showed that positive cell rates in the HZ of PCE(L) and PCE(H) fetal femurs were decreased in the dose-dependent manners (*P*<0.01, Figures 2c and f). These results suggest that the PCE can suppress terminal differentiation and apoptosis in the HZ.

### Effects of PCE on serum corticosterone and expression of Mig-6 and EGFR in the HZ of fetal rats

**Figure 3:**
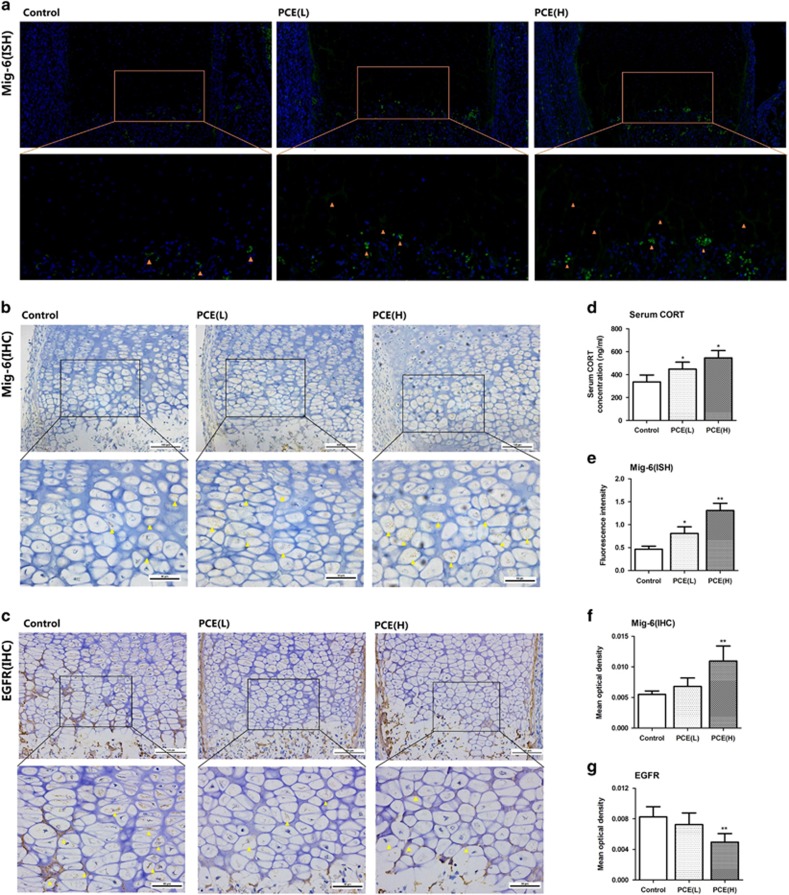
Effects of PCE on the expression changes of Mig-6 and EGFR in fetal long-bone hypertrophic chondrocytes. (**a**) ISH of Mig-6 in hypertrophic chondrocytes. (**b**) Immunostaining of Mig-6 in hypertrophic chondrocytes. (**c**) Immunostaining of EGFR in hypertrophic chondrocytes. (**d**) Serum corticosterone (CORT) concentration of fetal rats (ng/ml). (**e**) Quantification of Mig-6 ISH (fluorescence intensity). (**f**) Quantification of Mig-6 immunostaining (optical density). (**g**) Quantification of EGFR immunostaining (optical density). *n*=5 per group obtained from different litters. Three random fields/section for quantitative. Data are shown as the mean±S.D. *P<0.05, **P<0.01 *versus* control (ANOVA)

To investigate the mechanisms of PCE for suppressing of endochondral formation and the apoptosis of hypertrophic chondrocytes, the expression of Mig-6 and EGFR in the HZ of fetal rats are measured. We found that, serum corticosterone concentration of PCE(L) and PCE(H) fetal rats were higher than those of the control (*P*<0.05, Figure 3d). Further, through the ISH and IHC analysis, we found that the mRNA and protein expression of Mig-6 in the HZ of PCE(L) and PCE(H) groups were higher than those of the control (*P*<0.05, *P*<0.01, Figures 3a, b, e and f). Meanwhile, the protein expression of EGFR in the HZ of PCE(H) group was lower than that of the control (*P*<0.01, Figures 3c and g). These results suggest that the PCE increase serum corticosterone concentration, upregulate Mig-6 expression and inhibit EGFR expression in the HZ.

### Effects of corticosterone on the terminal differentiation and apoptosis in primary chondrocytes

**Figure 4:**
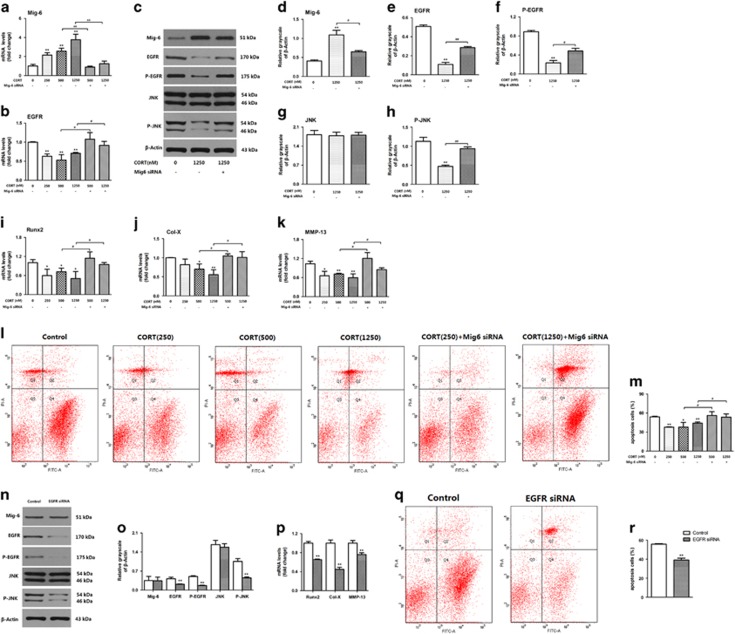
Effects of corticosterone (250–1250 nM) with/without siRNA (Mig-6, EGFR) for 48 h on rats primary chondrocytes terminal differentiation and apoptosis. (**a** and **b**) mRNA expression of mitogen-inducible gene 6 (Mig-6) and EGFR after corticosterone and Mig-6 siRNA treatment, (**c**) Protein expression of Mig-6, EGFR, phosphorylated EGFR (P-EGFR), c-Jun N-terminal kinase (JNK) and Phosphorylated JNK (P-JNK) detected by western blotting after corticosterone and Mig-6 siRNA treatment. (**d**–**h**) Quantification of Mig-6, EGFR, P-EGFR, JNK and P-JNK (relative grayscale). (**i**–**k**) mRNA expression of runt-related transcription factor 2 (Runx2), collagen type X (Col-X) and matrix metalloproteinases-13 (MMP-13) after corticosterone and Mig-6 siRNA treatment. (**l** and **m**) Apoptotic analysis detected by Annexin V/PI after corticosterone and Mig-6 siRNA treatment. (**n**) Protein expression of EGFR, JNK and P-JNK detected by western blotting after EGFR siRNA treatment. (**o**) Quantification of Mig-6, EGFR, P-EGFR, JNK and P-JNK (Relative grayscale). (**p**) mRNA expression of Runx2, Col-X and MMP-13 after EGFR siRNA treatment. (**q**) Apoptotic analysis detected by Annexin V/PI after EGFR siRNA treatment. Data are shown as the mean±S.D. of results from three experiments. *P<0.05, **P<0.01 *versus* control; ^#^*P*<0.05, ^##^*P*<0.01 *versus* CORT treatment group. (A-M, ANOVA; O-R, *t* test)

To further verify the mechanisms of Mig-6 and EGFR changes in the PCE fetal chondrocytes, primary chondrocytes were treated with different concentrations of corticosterone for 48 h, and then, the expression of the related genes were tested by RT-qPCR and western blot. We found that Mig-6 mRNA expression levels in chondrocytes treated with corticosterone (250, 500 and 1250 nM) were higher than that of the control in the concentration-dependent manners (*P*<0.05, Figure 4a). The expression levels of EGFR mRNA in chondrocytes treated with corticosterone (250, 500 and 1250 nM) were lower than that of the control (*P*<0.01, Figure 4b). After knockdown Mig-6 by small interfering RNA (siRNA) (*P*<0.01, Figure 4a), the expression of EGFR mRNA in Mig-6 siRNA and corticosterone (500 and 1250 nM) co-treated chondrocytes were higher than that of corticosterone (500 and 1250 nM)-treated chondrocytes (*P*<0.05, Figure 4b). Western blot showed that corticosterone (1250 nM) upregulated the expression of Mig-6 and downregulated the expression of EGFR, P-EGFR and P-JNK, whereas knockdown Mig-6 by siRNA partial reversal the expression of EGFR, P-EGFR and P-JNK (*P*<0.05, *P*<0.01, Figures 4c–h). These results indicated that corticosterone could upregulate the expression of Mig-6 and then inhibited the EGFR/JNK pathway expression in chondrocytes.

Simultaneously, we detected the mRNA expression of terminal differentiation marker genes and found that the mRNA expression levels of Runx2, MMP-13 and Col-X in corticosterone (250, 500 and 1250 nM)-treated groups were lower than those of the control (*P*<0.05, *P*<0.01, Figures 4i–k). After knockdown Mig-6, the mRNA expression levels of Runx2, MMP-13 and Col-X in corticosterone (500 and 1250 nM) and siRNA co-treated group were higher than those of the corticosterone (500, 1250 nM)-treated group (*P*<0.05, *P*<0.01, Figures 4i–k). Annexin V/PI results exhibited that the apoptosis rates of chondrocytes in corticosterone (250, 500 and 1250 nM)-treated group were lower than that of the control (*P*<0.05, Figures 4l and m). After co-processing with Mig-6 siRNA, the apoptosis rates in corticosterone (500 and 1250 nM) and siRNA co-treated groups were significant higher than that of corticosterone (500 and 1250 nM)-treated groups (*P*<0.05, Figures 4l and m). To verify whether the EGFR/JNK pathway regulate the terminal differentiation and apoptosis of HZ chondrocytes, we knockdown EGFR by siRNA and found that, EGFR knockdown lead to expression decrease of P-EGFR and P-JNK without effecting Mig-6 (*P*<0.01, Figure 4o). Meanwhile, the apoptosis decreased and terminal differentiation genes (Runx2, Col-X, MMP-13) expression downregulated (*P*<0.01, Figure 4p). These results suggested, corticosterone probably inhibit the terminal differentiation and inhibit the apoptosis of chondrocytes through upregulation of Mig-6 and consequently suppression of EGFR/JNK pathway.

### Effects of caffeine on terminal differentiation and apoptosis in primary chondrocytes

**Figure 5:**
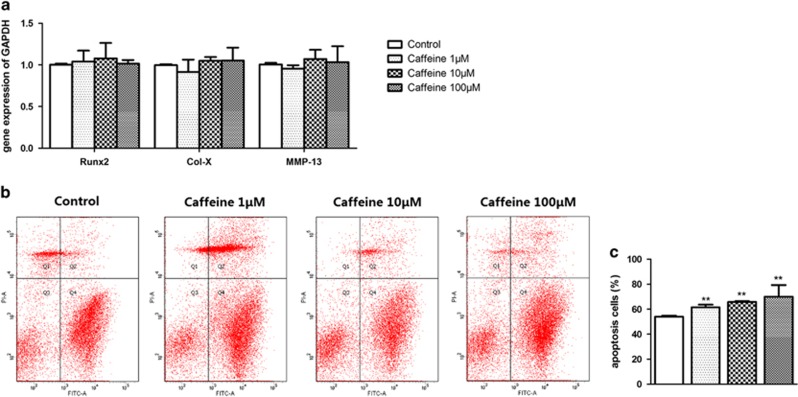
Effects of caffeine (0.1–100 *μ*M) on primary rat chondrocytes terminal differentiation and apoptosis. (**a**) mRNA expression of mRNA expression of runt-related transcription factor 2 (Runx2), collagen type X (Col-X) and matrix metalloproteinases-13 (MMP-13). (**b** and **c**) Apoptotic analysis by Annexin V/PI. Data are shown as the mean±S.D. of results from three experiments. *P<0.05, **P<0.01 (ANOVA)

To confirm whether the caffeine involved in PCE-caused the inhibition of terminal differentiation, the primary chondrocytes were treated with different concentrations of caffeine for 48 h. Then, the mRNA expression of Runx2, MMP-13 and Col-X were detected by RT-qPCR, and it was found that there were no significant differences between caffeine (1, 10 and 100 *μ*m)-treated and control groups (Figure 5a). However, the cell apoptosis in caffeine (1, 10 and 100 *μ*M)-treated cells was higher than that in the control in a concentration-dependent manner (*P*<0.05, *P*<0.01, Figures 5b and c). These results indicated that caffeine induced the apoptosis of chondrocytes but had no effect on their terminal differentiation.

## Discussion

Epidemiological evidence reveals that a caffeine intake of 300 mg/day during pregnancy was associated with an increased risk for IUGR.^20^ Although caffeine intake during pregnancy is different in dose and source, it is relevant to increasing the risk of a low birthweight and premature delivery.^3, 4, 21^ Our previous studies demonstrated that PCE could induce rat IUGR and multi-organ developmental toxicities, and a PCE-induced rat IUGR model was stably established with 30–120 mg/kg day caffeine at GD9-20 in our laboratory.^8, 9, 19, 22, 23^ According to the dose conversion between humans and rats (human:rat, 1:6.17 by body surface area comparison),^24^ 30 mg/kg day caffeine exposure in rats is equal to 2.3–3.3 cups of caffeine consumed by a human per day (each cup contains 100–150 mg caffeine), which can been achieved in our daily life. Therefore, we believe that this PCE-induced rat IUGR model used in the present study was rational for clarifying long-bone development toxicity and its molecular mechanism.

Endochondral ossification is a critical process in long-bone development. Chondrocytes undergo proliferation, hypertrophy and apoptosis, and the cartilage matrix surrounding hypertrophic chondrocytes left behind provides a scaffold for osteoblasts laying down a true bone matrix.^25^ The terminal differentiation and apoptosis of hypertrophic chondrocytes is critical to endochondral ossification.^26, 27^ There are a series of key regulators during hypertrophic chondrocytes terminal differentiation, such as Runx2, Col-X and MMP-13.^28, 29^ The function of Runx2 in hypertrophic chondrocytes is regulates the OPG-RANKL signaling required for cartilage resorption.^30^ Col-X is widely known for its important role during the mineralization of hypertrophic chondrocytes.^31^ As a member of matrix metalloproteinases, MMP-13 participants in extracellular matrix degradation and the apoptosis of chondrocytes, thus providing space for endochondral ossification.^32^ In the present study, we found the length of femur and primary ossification center were apparently shortened in PCE fetus, which suggesting the retardation of long-bone development. Through microscopic observation, we found that the HZ of PCE fetal femur significantly broadened. The PZ and PHZ has no significant change and we confirmed this through Ki67 and p57 (early differentiation maker) analysis by IHC. Moreover, the chondrocytes in the hypertrophic zone showed decreasing apoptosis and low expression levels of Runx2 and Col-X. These results suggested that the inhibition of terminal differentiation and apoptosis caused by PCE led to a broadened growth plate HZ and finally made fetal femurs shorter.

Increases in both systemic glucocorticoid treatment and endogenous glucocorticoid can bring about child growth retardation.^33, 34^ Research showed that long-term exposure to a higher level of glucocorticoid induces abnormal fetal development.^35^ Glucocorticoid has a very important role in long-bone growth plate development.^36^ Our previous study indicated that PCE made fetal rats over-exposed to maternal glucocorticoid, which related to downregulation of 11*β*-hydroxysteroid dehydrogenases 2 (11*β*-HSD2) in the placenta, whereas 11*β*-HSD2 oxidizes and inactivates glucocorticoids.^9^ In the present study, we confirmed the higher levels of fetal serum corticosterone after 30 and 120 mg/kg day caffeine administration. Through treating primary chondrocytes with different concentrations of corticosterone *in vitro*, we found the reduction in the terminal differentiation markers (such as Runx2, Col-X and MMP-13), accompanied by apoptosis abatement. Caffeine can go through the placenta into the fetal body. It is a worthwhile concern whether caffeine may have a direct effect on chondrocyte terminal differentiation. It is interesting that the *in vitro* experiment showed that different concentrations of caffeine could induce chondrocyte apoptosis without significant changes in terminal differentiation markers. This indicates that the effects of caffeine on chondrocytes *in vitro* were not the same as those in the animal experiment. These results suggested that corticosterone, rather than caffeine, mediated PCE-induced suppression of terminal differentiation and apoptosis in the HZ.

As we know, the EGFR signaling pathway takes part in several processes in bone and cartilage development.^14^ EGFR knockdown mice have a broadened HZ, reduced osteoclast recruitment and finally poor osteogenesis,^15^ which reveals that the EGFR signaling pathway regulates chondrocyte terminal differentiation in endochondral ossification. Literatures suggested that,^37^ EGFR mediate cell apoptosis mainly through c-Jun N-terminal kinase (JNK) signaling, suggesting that JNK signaling may be involved in the regulation of corticosterone on chondrocyte apoptosis. It has verified that Mig-6 can be recruited and bind with the EGFR kinase domain to inhibit its phosphorylation and downstream signaling pathway.^17^ Already know, Mig-6/EGFR participates in the regulation of articular chondrocytes metabolism.^38^ Moreover, a high glucocorticoid level can suppress basic and EGF-induced EGFR phosphorylation through upregulating Mig-6.^18^ Therefore, high levels of glucocorticoid may affect terminal differentiation and apoptosis of chondrocytes and development of long bone through activating Mig-6 to downregulate the EGFR/JNK signaling pathway. In our study, the fetal HZ chondrocytes of the PCE group showed a high-expression level of Mig-6 and a low expression level of EGFR. Meanwhile, primary chondrocytes treated with different concentrations of corticosterone *in vitro* exhibited similar changes in Mig-6 and EGFR/JNK pathway expression levels with the HZ chondrocytes in the PCE group *in vivo*. Moreover, the downregulation of cell apoptosis and Runx2, MMP-13 and Col-X genes expression by corticosterone can partially reversed after knockdown Mig-6. Knockdown EGFR can lead to the decreased expression of P-EGFR and P-JNK; meanwhile inhibit the cell apoptosis rate and terminal differentiation marker genes expression of chondrocytes without effecting the expression of Mig-6. Therefore, our results suggested that high levels of glucocorticoid activated Mig-6 expression and the subsequently suppressed EGFR/JNK signal, ultimately influenced terminal differentiation and resulted in long-bone development retardation.

Barker and Hales put forward that a fetus would make ‘adaptation changes’ to benefit itself to adapt to undesirable intrauterine environments.^39^ The adaptions of the fetus are also called the ‘thrifty phenotype’. Our previous studies indicated that the PCE-induced IUGR offspring have multi-organ developmental toxicities and related disease susceptible. For example, some organs exhibit functional development enhancement, such as the increased hepatic lipid synthesis in fetuses and enhanced susceptibility to non-alcoholic fatty liver disease in adults.^23^ However, most organs exhibit functional development inhibition, such as the inhibition of the matrix synthesis of articular cartilage in fetuses and enhanced susceptibility to osteoarthritis in adults,^40^ inhibition of steroid synthesis of the adrenal gland in fetuses and enhanced susceptibility to hyperadrenocorticism in adults,^19^ and podocyte dysplasia of kidneys in fetuses and enhanced susceptibility to glomerulosclerosis in adults.^41^ High intrauterine glucocorticoid levels bring about fetal multi-organ structural and functional abnormities through regulating a series of neuroendocrine developmental programs.^42, 43^

Specific to PCE-induced long-bone development, in the present study, the high fetal corticosterone level (rather than caffeine) could suppresses terminal differentiation of the HZ through the Mig-6 and EGFR/JNK signal, ultimately resulting in the retardation of long-bone development. The purpose of maternal glucocorticoid over-exposure is probably to control and sacrifice long-bone growth and development in order to ensure the development of other important organs (e.g., liver) and help fetuses to pass the crisis and survive in adverse environments. Therefore, we believe that, in the present study, the glucocorticoid-induced retardation of endochondral ossification of long bone is a concrete manifestation of ‘thrifty phenotype’ programming in IUGR individuals caused by PCE.

## Conclusion

**Figure 6:**
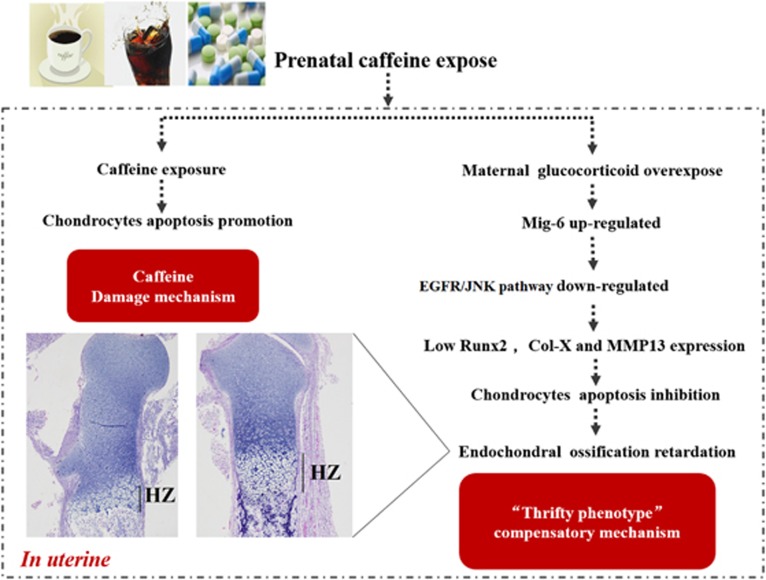
The proposed schematic model of the present study. Col-X, collagen type X; EGFR, epidermal growth factor receptor; JNK, c-Jun N-terminal kinase; Mig-6, mitogen-inducible gene 6; MMP-13, matrix metallopeptidase 13; Runx2, runt-related transcription factor 2

In summary, our study confirmed that the PCE could cause the retardation of long-bone endochondral ossification in the female IUGR fetal rats, and its underlying mechanisms are associated with a high level of glucocorticoid rather than caffeine. As Figure 6 shows, high glucocorticoid level induced by PCE in a fetus could increase Mig-6 expression, subsequently suppressing the EGFR/JNK signal and cell apoptosis, finally leading to the inhibition of chondrocyte terminal differentiation and retardation of endochondral ossification. This study not only clarified the molecular mechanism of PCE-induced long-bone developmental toxicity but also provided a foundation for revealing maternal high glucocorticoid programming fetal osteoporosis susceptibility and understanding the international hot issue of DOHaD.

## Materials and methods

### Drugs and reagents

Caffeine (No. C0750) was purchased from the Sigma-Aldrich (St. Louis, MO, USA). Diethyl ether (No. 10009318) was obtained from the Sinopharm Chemical Reagent (Shanghai, China). The rat corticosterone enzyme-linked immunosorbent assay (ELISA) kit (No. EC3001-1) was obtained from Assaypro (Saint Charles, MO, USA). A primary antibody such as anti-Ki67 (No. ab15580) was purchased from Abcam (Cambridge, UK). Polyclonal antibodies for P57 (No. sc-71824), Mig-6 (No. sc-137154), phosphorylated EGFR (P-EGFR) (No. sc-57541), and runt-related transcription factor 2 (Runx2) (No. sc-390715) were obtained from Santa Cruz Biotechnology (Santa Cruz, CA, USA). A primary antibody such as EGFR (No. BM4009), JNK (No. BM4329) and phosphorylated JNK (P-JNK) (No. BM4380) were obtained from BOSTER Biological Technology (Wuhan, China). Terminal deoxynucleotidyl transferase dUTP nick-end labeling (TUNEL) Detection Kit (No. 11772465001) and Fluorescein TUNEL Detection Kit (No. 11684795910) was from Roche Molecular Biochemicals (Indianapolis, IN, USA). Lipofectamine 3000 Transfection Reagent (No. L3000015) and the SYBR Select Master Mix (No. 4472980) were purchased from Applied Biosystems by Thermo Fisher Scientific (Waltham, MA, USA). Mig-6 and EGFR siRNA were purchased from the GenePharma Pharmaceutical Technology (Shanghai, China). Annexin V-FITC/PI kit (No. 401002) was purchased from BestBio Biotechnology (Shanghai, China). The TRIzol reagent kit was obtained from Omega Bio-Tek (Doraville, GA, USA). Reagents used for cell culture including Hank's balanced salt solution (HBSS), phosphate-buffered saline (PBS), and dimethyl sulfoxide (DMSO), Dulbecco's Modified Eagle Medium:Nutrient Mixture F-12 (DMEM/F-12), streptomycin, penicillin and trypsin were purchased from Invitrogen by Thermo Fisher Scientific. Reverse transcription and real-time quantitative PCR (RT-qPCR) kits (No. DRR047A) were purchased from the Takara Biotechnology (Dalian, China). All of the primers were synthesized by the Sangon Biotech (Shanghai, China). Other chemicals and agents were of analytical grade.

### Animals and treatment

Animal experiments were performed in the Center for Animal Experiments of Wuhan University (Wuhan, China), which is accredited by the Association for the Assessment and Accreditation of Laboratory Animal Care International (AAALAC International). The Committee on the Ethics of Animal Experiments of the Wuhan University School of Medicine approved the protocol (permit number: 14016). All animal experimental procedures were performed in accordance with the Guidelines for the Care and Use of Laboratory Animals of the Chinese Animal Welfare Committee.

Specific pathogen-free Wistar rats (No. 2012-2014, certification number: 42000600002258, license number: SCXK (Hubei)) weighing 209±12 g (females) and 258±17 g (males) were obtained from the Experimental Center of the Hubei Medical Scientific Academy (Wuhan, China). Animals were housed in metal cages with wire-mesh floors in an air-conditioned room under standard conditions (room temperature: 18–22 °C, humidity: 40–60%, light cycle: 12 h light-dark cycle and 10–15 air changes per hour) and allowed free access to rat chow and tap water. All rats were acclimated 1 week before experimentation. The IUGR model based on our previous study,^25^ which proceeded according to the following steps: two female rats were placed together with one male rat overnight in a cage for mating. The appearance of sperm in vaginal smears confirmed mating, and the day of mating was taken as the gestational day (GD) 0. Pregnant rats were transferred to individual cages and then randomly divided into the control, PCE(L) and PCE(H) groups. From GD9 to GD20, the PCE(L) and PCE(H) groups were intragastrically administered 30 and 120 mg/kg day caffeine, respectively, and the control group was administered the same volume of distilled water. On GD20, the pregnant rats were anesthetized with diethyl ether, and those rats with litter sizes of 8–14 were considered qualified. The female fetuses from each litter were decapitated immediately to collect serum, and the serum samples of female fetuses from each litter were pooled and immediately frozen at −80 °C for subsequent analyses. Portions of the female fetal unilateral femurs obtained from different litters were fixed in 4% paraformaldehyde for morphological observation. In *in vivo* experiment of this study, we select 5 (*n*=5) female fetal rats obtained from different litters per group for experiment.

### Serum corticosterone concentration analysis

The concentration of serum corticosterone was measured by an ELISA kit, following the manufacturer's protocols. The intra-assay and inter-assay coefficients of variation for corticosterone determination were 5 and 7.2%, respectively.

### Histological analysis, immunohistochemical analysis and ISH

The femur was fixed in 4% paraformaldehyde solution overnight and processed with the paraffin sectioning technique. Tissues were cut into 5 *μ*m thickness sections prepared for morphological testing.

Immunohistochemistry analysis according to the following steps: after antigen retrieval, bone sections were blocked in 5% blocking serum at room temperature for 30 min and probed with primary antibodies overnight at 4 °C. Sections were incubated with primary antibodies including rabbit anti-Ki67 (1:100 dilution), rabbit anti-P57 (1:100 dilution), rabbit anti-Runx2 (1:100 dilution), rabbit anti-Mig-6 (1:100 dilution), and rabbit anti-EGFR (1:200 dilution). Then, they were incubated with a biotinylated secondary antibody, followed by incubation with an avidin-biotinylated horseradish peroxidase complex solution according to the manufacturer's directions. Finally, the peroxidase activity was determined with a diaminobenzidine (DAB) staining kit. Apoptosis detection was carried out using the TUNEL and Fluorescein TUNEL system from Roche Molecular Biochemicals (Indianapolis, IN, USA) according to the manufacturer’s directions. Three random field/section were selected for quantitative analysis. Positive cell rate and optical density of each image counted by the Photo Imaging System (Nikon H550S, Tokyo, Japan).

ISH of collagen type X (Col-X) and Mig-6 performed in BOSTER Biological Technology according to the following steps: after dewaxing, the sections immersed in pepsin diluted with 3% saline citrate for 3–30 min to expose mRNA nucleic acid fragments. Then, after post-fixation with 1% paraformaldehyde in PBS (pH=7.2–7.6), the sections were added to 20 *μ*l of pre-hybridization solution for 2–4 h at 38–42 °C and added to 20 *μ*l of hybridization solution overnight at 38–42 °C successively. After washing and blocking (for 30 min at 37 °C), the slides were incubated with biotinylated mouse anti-digoxigenin, followed by SABC (for 20–30 min at 37 °C) and biotinylated peroxidase (for 20 mi at 37 °C) successively. Finally, the peroxidase activity was determined with a DAB staining kit. Three random field/section were selected for quantitative analysis. Fluorescence density of each image counted by the Olympus AH-2 light microscope (Olympus, Tokyo, Japan).

### Cell culture and treatment

The chondrocytes digested from the knees of newly born Wistar rats were plated at a density of 2 × 10^5^ cells per well in 6-well plates in medium (DMEM/F-12 medium with 5% fetal bovine serum, 100 mg/ml streptomycin and 100 U/ml penicillin). Chondrocytes were treated with corticosterone (0, 250, 500 and 1250 nM) and caffeine (0, 0.1, 1, 10 and 100 *μ*M) for 48 h, and then harvested for mRNA and protein analysis. To detect the apoptosis of chondrocytes, part of chondrocytes were incubated with 400 *μ*l of 1 × Annexin V binding buffer, followed by 5 *μ*l of Annexin V-FITC dye solution (for 15 min at 2–8 °C) and 10 *μ*l of PI dye solution (for 5 min at room temperature) in a non-light environment. In *in vitro* experiment of this study, all experiment repeated three times independently.

### siRNA knockdown

Cell preparation: The day before transfection, chondrocytes were seeded in 6-well plates at 2 × 10^5^ per well to maintain the cell abundance at 70% when transfection. Transfection solution preparation: Dilute 40 pmol of siRNA oligo to 250 *μ*l of DMEM serum-free medium to form diluted siRNA; dilute 10 *μ*l of Lipofectamine reagent (Thermo Fisher) to 250 *μ*l of DMEM medium to from RNAi-Mate reagent; mix the diluted siRNA and the RNAi-Mate reagent and placed at room temperature for 20 min to form the siRNA/lipofectamin complex. Add 500 *μ*l of siRNA/lipofectamin complex to the wells evenly and incubate in a 37 °C incubator for 8–12 h to complete the transfection.

### Total RNA extract and RT-qPCR

The total RNA of primary chondrocytes was isolated using TRIZOL reagent following the manufacturer’s protocol. The total RNA was reverse transcribed using a first strand cDNA synthesis kit. The cDNA was amplified using a one-step RT-qPCR reaction. The primer sequences for the rats and annealing temperature are shown in Figure 6 and Table 1.

### Western blot

Primary antibodies used were anti-Mig-6 (1:2000), anti-EGFR (1:500), anti-p-EGFR (1:2000), anti-JNK (1:2000), anti-p-JNK (1:1000) and anti-*β*-actin (1:5000). Secondary antibodies used were goat anti-rabbit HRP for Mig-6 (1:3000), goat anti-mouse HRP for p-EGFR (1:3000), HRP-Goat anti-Rabbit for EGFR, JNK, p-JNK (1:10 000) and goat anti-mouse HRP for anti-*β*-actin (1:5000).

### Statistical analysis

SPSS 17 (SPSS Science, Chicago, IL, USA) and Prism 6.0 (GraphPad Software, La Jolla, CA, USA) used to perform data analysis. Quantitative data expressed as the mean±S.D. The data in *in vivo* experiments with different doses PCE and in *in vitro* experiment with different drug concentrations analyzed using one-way analysis of variance (ANOVA) with *post hoc* test for multiple comparisons. In part of the *in vitro* experiment, we used unpaired, two-tailed Student's *t*-tests for comparisons between control and EGFR knockdown groups. Statistical significance was defined as *P*<0.05.

## Publisher’s Note

Springer Nature remains neutral with regard to jurisdictional claims in published maps and institutional affiliations.

## Figures and Tables

**Table 1:** Primer used for real-time quantitative polymerase chain reaction

**Genes**	**Forward primer**	**Reverse primer**
*Mig-6*	CCTACAATCTGAACTCCCCTG	AGCTTGACTTTGGAGATGGAC
*EGFR*	ACCAGCAGGACTTCTTTCCCA	TAAACTCACTGCTTGGCGGTG
*Runx2*	GCCACCTTCACTTACACCCC	CGCTGACGAAGTACCATAGTAGAG
*MMP-13*	TGACCTGGGATTTCCAAAAGAG	GTCTTCCCCGTGTCCTCAAA
*Col-X*	GCGCATCATGAATCTCGTTT	GGGTTGTGTGAACCATGGAG
*GAPDH*	GCAAGTTCAACGGCACAG	GCCAGTAGACTCCACGACA

Col-X, collagen type X; EGFR, epidermal growth factor receptor; GAPDH, glyceraldehydes 3-phosphatedehydrogenase; Mig-6, mitogen-inducible gene 6; MMP-13, matrix metallopeptidase 13; Runx2, runt-related transcription factor 2.
